# Finland’s New Children’s Hospital and resurgent charity in a Nordic post-welfare state

**DOI:** 10.1136/medhum-2024-013128

**Published:** 2025-02-08

**Authors:** Henni Alava, Janette Lindroos, Arvi Pihlman

**Affiliations:** 1 Unit of Social Research, Tampere University, Tampere, Finland

**Keywords:** paediatrics, medical anthropology, Child health, Health policy, cultural studies

## Abstract

Finland’s New Children’s Hospital (NCH) opened in 2018 after a high-profile charity campaign. Through an analysis of the campaign, we illustrate how debates about children’s hospitals and charities are simultaneously shaped by universal debates over the ownership and funding of healthcare services, and by the particularities of historical context and local institutional arrangements. Such debates also draw from and contribute to shaping cultural repertoires, particularly shared beliefs and values concerning children, the state and charity. In Finland, the NCH marked a break from an established model of publicly funded hospitals, and a return to the pre-welfare state era when charities played a role in healthcare. The campaign thus generated substantial public debate, in which we identify three core claims: (1) Hospitals must be built with public funding and oversight. (2) Children are suffering because politicians have failed, and a new model is needed. (3) The NCH further strengthens Finland’s excellence in paediatric healthcare and promotes health technology exports. Over the course of the campaign, critique faded away, and the second and third lines of argument came to dominate public debate. Through a reflection on the historically changing relations between state, charity and children that shaped what we conceptualise as the NCH assemblage, we show how clichéd cultural tropes naturalised the political shift toward a post-welfare state that is embedded in the NCH campaign. As citizens without voter rights, children are exceptionally easy for politicians to sidestep when allocating funds. Yet, as what Sara Ahmed describes as ‘objects of feeling’, children are also exceptionally potent targets for charity, who, in the NCH case, came to serve as tools for the neoliberal disassembling and reassembling of healthcare services in Finland.

## Introduction

 The 2018 opening of the New Children’s Hospital (NCH) in Finland’s capital city, Helsinki, drew an end to a public debate and charity campaign that had swept across the small Nordic nation. Only 6 years earlier, public outrage at the outdatedness of the old Children’s Clinic (Lastenklinikka), and the failure of politicians to prioritise paediatric care, had triggered the establishment of the New Children’s Hospital Foundation (NCHF, Uuden lastensairaalan tukisäätiö) to lobby for funding for the new hospital, as Finnish legislation prohibits public institutions from receiving donations. The foundation owns and maintains the hospital and rents the premises to the local hospital district, HUS, which will either take over ownership or stay on as a tenant as soon as the NCH’s construction loans are repaid. The NCH charity campaign inspired ordinary Finns, alongside political, cultural and business elites, to take part, contributing altogether 36.5 million euros toward the hospital’s construction. Branding itself as interested only in the well-being of sick children and their families, the campaign was an unprecedented success, and the critical public debate it attracted dissipated quickly following a brief flutter of protest. In this article, we argue that the campaign for the NCH, and its culmination in the completed hospital, symbolises a shift in Finnish public perceptions of the role of charity in healthcare provision.

A key concern of this special issue is the exceptional role charity plays in children’s hospitals, and children play for charities. As (SI Introduction) shows, children’s hospitals are uniquely well-placed as rallying points for donations, and charitable organisations have historically been pivotal in establishing them. This is also the case in Finland, where the predecessors of the NCH include the public children’s hospital Lastenklinikka (established in 1893) and Lastenlinna, which originally opened in 1917 as a charitable enterprise before being merged with the public system in 1997. In a sense, then, the charity-funded NCH signifies a return to the past. Yet, we argue that it also signals the ongoing reassembling and transformation of Finnish healthcare in what critical scholars describe as Finland’s post-welfare state (see Baeten *et al* 2016; Honkasalo *et al* 2022).

A point worth highlighting in relation to other cases discussed in this issue is that charity holds a very different place in the Finnish collective imaginary than in many European contexts. Finnish charities have a particularly negligible role in healthcare financing, and Finnish public debate on healthcare pivots on a bifurcated public-private axis, wherein ‘charity’ is most often intuitively grouped under ‘private’ or dismissed altogether. A recent edited volume, *The Nordic Economic, Social and Political Model. Changes in the 21st Century* (Koivunen *et al* 2021), is indicative of this underlying pattern of thought and social infrastructure, as the book contains no reference to ‘charity’ at all. Relatedly, tax-deductible donations are currently limited to universities (medical faculties being central recipients) and the arts, and while the current government plans to expand this, social and health sector non-governmental organisations are to be excluded (Paananen 2024).

Our analysis of the NCH underlines our key contention that debates about children’s hospitals and charities are simultaneously shaped both by universal debates over the ownership and funding of healthcare services, and by the particularities of historical context and local institutional arrangements. Such debates also draw from and contribute to shaping cultural repertoires, particularly shared beliefs and values concerning children, the state and charity. We bring together literatures on these themes under a theoretical approach that hinges on the notion of assemblage (Duff 2023; Wahlberg 2022); that is, we trace some of the key lines (Youdell and McGimpsey 2015) that came together to produce the hospital, as well as the ways in which the completed hospital—the buildings, administrative structures, bank loans and people that constitute it—in turn shape public imaginaries about the future of children, their health and the welfare state to come.

Three core arguments emerged in our analysis of the public debate surrounding the NCH campaign. The first argument, that hospitals must be built with public funding and oversight, was prominent at the very beginning of the campaign, but faded away over time as the second argument took centre stage. It held that children are suffering because politicians have failed, and thus a new model is needed. The third line of argument came to the fore once debates over the funding mechanism faded to the background and spoke for the construction of the NCH on the grounds of Finland’s excellent track record in paediatric healthcare and in health technology development.

These arguments were interwoven with appeals to cultural values in ways that contributed to reifying idealised understandings of ‘Finnishness’. The appeal to certain cultural tropes—ones poignantly identified in the front-page advertisement that launched the campaign in Finland’s leading daily *Helsingin Sanomat*, as *sisu* (grit), *talkoot* (cooperative work), *sydän* (heart) and *mutkat suoriksi* (straightened curves)—served to naturalise the political shift that the NCH campaign represented. The NCH’s current position in Finnish society—and its disappearance from political debate—serves as evidence of the campaign’s partaking in the making of an increasingly neoliberal assemblage of healthcare in Finland. The employment of clichéd cultural tropes in political debate illustrates how the rise of charity not only *masks* the neoliberalisation of society, but, as Clarke and Parsell (2022) suggest, actually contributes to reassembling the social. To use Ahmed’s (2004) terms, children functioned as particularly potent ‘objects of feelings’ in this process: they drew in charity funds and created a sense of urgency for a campaign that advanced the neoliberalisation of cultural norms and political imaginaries concerning healthcare provision.

## Paediatric care and changing welfare models in Finland

Finland’s first 22-bed children’s hospital, which came to be known as Lastenklinikka*,* was founded in Helsinki in 1893 to support paediatric medical education (Tuuteri 1993, 11). At the time, Finland’s infant mortality rate was considerably higher than in neighbouring Sweden and Norway, although lower than in other parts of the Russian Empire (Tuuteri 1993, 19) to which Finland belonged, gaining its independence in 1917. By this period of capitalist industrialisation and urbanisation, official social policy had started to take shape, yet for the first half of the 20th century, welfare and care relied largely on non-state actors: from families and communities to philanthropic charities and social movements (Markkola 2005). As in other parts of Europe (eg, Alexander 2009), these earlier forms of collective care and civic activism laid the groundwork for the welfare state that formed later.

Finland’s infant mortality rate dropped from 153.1 deaths per 1000 live births in 1900, to 96.7 in 1920, to 29.7 in 1955 (Statistics Finland 2024). In addition to rising standards of living, this was due to improved paediatric care in which public-philanthropic collaboration played a key role, specifically the care of two pioneers of Finnish health practice: chief physician Arvo Ylppö, and nurse Sophie Mannerheim. In 1918, Mannerheim founded Lastenlinna, first as a shelter for lonely mothers, then as a children’s hospital run by the child protection and welfare-focused charity Mannerheimin lastensuojeluliitto (MLL). Lastenlinna became the launching site of the maternal and child health centre (*neuvola*) network, which was to become a cornerstone of Finnish public health. The charity-run Lastenlinna worked in parallel with the public children’s hospital, Lastenklinikka. The history of these institutions was interestingly replayed in the NCH saga. In an article published in the national daily *Helsingin Sanomat* (HS) in 2013, one of the old public children’s hospital nurses, aged 92 at the time of the interview, recalled the immediate post Second World War years, when the hospital badly needed renovation: ‘The situation (now) is hilariously similar to then. Newspaper articles have exactly the same tone. Good people thought that money should be found for the hospital, and bad financial people said there was no money’ (HS, 31 August 2013). In the decades that followed, rapid industrialisation, economic growth and tripartite negotiations between the labour movement, industry and centre-left political elites led Finland on a road towards the Nordic welfare model, characterised by a strong state and broad tax-financed welfare benefits and services (Christiansen and Petersen 2001, 154). Subsequently, the philanthropic roots of Finnish paediatric healthcare were also undone, and Lastenlinna hospital transitioned from MLL to Kuntainliitto (Union of Municipalities) in 1968, and then to Helsinki University Hospital in 1997.

Although Finland is often seen as the weakest representative of the Nordic model (Kuisma 2017, 435), by the 1980s, its public expenditures matched those of its Nordic neighbours (Kokkonen *et al* 2018, 36). The 1960s to the 1980s is considered the ‘golden age’ of Finland’s welfare state (eg, Alanko 2015), but the era also saw the creeping embrace of neoliberal ideology among leading politicians and public officials (Wuokko 2021, 677). Liberalisation of Finland’s financial sector, accompanied by a downturn in Western export markets and the collapse of the Soviet Union—which had been Finland’s important trade partner—led to an economic and social crisis in the early 1990s, made even worse by harsh austerity measures (Kokkonen *et al* 2018, 36–37). The 1990s depression marked a turning point in state policy from welfare towards competitiveness (Wuokko 2021, 677). Service outsourcing and marketisation has allowed third sector actors to regain more of the space they had prior to the heyday of the welfare state (Pihlman 2025). What is unparalleled in the recent transformation, however, is the role played by the international medical industrial complex (Honkasalo *et al* 2022; Illich 1979) and venture capitalism in shaping the contours of health and illness.

In Finland, the vast majority of tertiary care is provided in public hospitals. However, municipalities, recently federated into well-being services counties, have the right to arrange primary and secondary healthcare as they see fit, and recent years have seen the rapid outsourcing of public health services to private companies (Saltman and Teperi 2016). These companies also offer employer-funded occupational health services, as well as basic and specialist health services to those who can afford to pay for them, or for private health insurance. All these services are subsidised by the Social Insurance Institution of Finland (KELA). Finnish parents increasingly consider public healthcare insufficient, and health insurance has become a taken-for-granted part of a middle-class lifestyle (Lehtonen 2017; Sointu *et al* 2021); approximately half of those aged 4 and below now have private healthcare insurance (Paju *et al* 2023), although in cases of serious illness, care is routinely provided in public hospitals.

Although civic associations have complemented public services in the past, philanthropic charity has been marginal and seen as contrary to the welfare state’s principles (Wijkström 2011, 36). Neoliberal restructuring has changed this, with charity gaining prominence in welfare service provision (Wijkström 2011, 45). This reflects both the return of pre-welfare traditions and a shift towards business-oriented practices in care provisioning. While charities played a role in setting up healthcare infrastructures in the late 19th and early 20th centuries, their role has recently been marginal. Two exceptions stand out: private donations for medical research and targeted donations by charities for specific needs in public hospitals, such as incubators for neonatal intensive care units.

By the early 2000s, the old Lastenklinikka, which provided care for children in the Helsinki metropolitan area, was in poor condition. The building dated back to 1946, and it no longer met modern requirements and, despite numerous repairs, the hospital district considered it clear that a new building was needed (Heikkilä 2020). In the 1990s, an association, Lastenklinikan Tuki ry*,* had already been established to rally funding for a new hospital, but the association became mired in controversy and went bankrupt in 1993 after the chairperson was convicted of an accounting offence. Three years later, a new organisation, Lastenklinikan kummit (Godparent’s of the Children’s Clinic), was established; aided by celebrities, it continues to gather funds to support existing public children’s hospitals throughout Finland. Despite the attempts of such groups, and the views of the hospital’s administrators, the Helsinki children’s hospital was repeatedly deprioritised in public funding decisions.

In the early 2010s, things came to a head. As narrated in a documentary produced by the hospital district and Finland’s Foundation for Paediatric Research, *New Children’s Hospital – A Gift for the Children of Finland* (Cityportal 2019), paediatrician and Director of Operations at the Children’s Clinic, Jari Petäjä, joined forces with attorney Tuomas Aho to assemble prominent individuals, such as Finland’s former president Martti Ahtisaari, to advance the fundraising efforts of the NCH. The appointed head of fundraising was Anne Berner, a prominent business executive who, 2 years after launching the NCH fundraiser, was elected to parliament and appointed Minister of Transport and Communications.

Since the public sector in Finland is not allowed to campaign for charity funding, the New Children’s Hospital Support Association 2017 (Uusi lastensairaala tukiyhdistys 2017 ry) was established in 2012 to organise fundraising efforts. Soon after, in January 2013, the New Children’s Hospital Foundation (NCHF) was founded to oversee the construction of the hospital. To facilitate the practical implementation of the construction, it established a real estate company (Kiinteistö Oy Uusi Lastensairaala), which is owned by the NCHF, with no provision for additional ownership. The foundation owns the NCH property, and leases it to the well-being services county of the Helsinki region (HUS), which is responsible for the building’s maintenance. While doubts were raised in public debate about this ownership arrangement at the time of NCH campaign, the NCH publicly emphasises that the arrangement does not yield profit for the foundation; rather, rent covers the portion of the loan used to finance the hospital. On the NCH website (Uuden Lastensairaalan Tukisäätiö 2024), it is stipulated that the building will be transferred to HUS without compensation once the 30-year lease comes to an end. However, the lease contract, which we received a copy of from the NCHF when doing background research for this article, also contains the option that once the lease ends, HUS can stay on as a tenant of the foundation. The NCH is the first public hospital to be funded and owned in this way in Finland.

In February 2013, the NCH Support Association 2017 launched the NCH fundraising campaign under Berner’s leadership with the goal of raising 30 million euros. The campaign was advertised nationwide in various media outlets and covered in television and print media, featuring stories about donations made by companies and individuals. Taking their cue from ‘Live Aid’ events held since the 1980s to gather funds for various charities, a song titled *Lohtu* (comfort) was released by top artists in 2014, and the fundraising culminated in a ‘Live Aid New Children’s Hospital 2017’ concert, broadcast with great hype and high viewer ratings on national television in the summer of 2015. While the goal was achieved in August 2014, fundraising continued until the expiration of the permit in July 2015. The funding for the NCH ultimately consisted of three parts: 36.5 million euros in donations, 80 million euros of public funding (split 50/50 between the state and the hospital district), and a market loan of 66.3 million euros, taken out by the NCHF.

The goal was to open the NCH in 2017 to mark Finland’s centenary of independence, but the construction was not completed until the following year. Since opening in September 2018, the NCH has delivered specialised inpatient and outpatient care as well as night-time and weekend emergency services to young patients in the metropolitan area; it has also provided certain highly specialised services, such as paediatric heart surgery and organ transplants, to young patients throughout Finland. In the following section, we introduce the conceptual framework we employ to analyse the campaign for the NCH and the hospital resulting from it.

## Affective dis/assemblings of charity, children and the welfare state

Finnish political language captures the ongoing process of neoliberalisation, by which we refer to the shifting of responsibilities from public to private entities, the reduction of public funding and an increased reliance on market mechanisms and voluntarism: the term ‘welfare society’ (*hyvinvointiyhteiskunta*) is increasingly used in place of ‘welfare state’ (*hyvinvointivaltio*)—typically without speakers specifying what the revised terminology entails (Julkunen 2001). From an assemblage perspective, however, neoliberalisation is not identical across every context, rather, its form depends on the relations between material, discursive, human and more-than-human elements in a given locus. At the heart of neoliberalism, Muehlebach (2012) argues, alongside the purported ‘rationality’ and ‘effectiveness’ of the market, is the charitable citizen. Neoliberal social actors—both state and non-state—imagine citizens ‘to be animated by affect rather than intellect, by the capacity to feel and act on these feelings’ (Muehlebach 2012, 8), whereby solidarity is outsourced from the polity to civil society and individuals. Clarke and Parsell (2022, 317), adapted Muehlebach’s analysis to social services in the UK, arguing that the neoliberal resurgence of charity has excluded recipients from reciprocal relationships, depoliticised problems by obscuring their structural causes and normalised post-political modes of governance. Similar processes can be identified across contexts and scales.

In his analysis of international humanitarianism, Fassin (2013) interpreted the circulation of images and narratives of suffering and help as essential to the global reassembling of the social. Such stories provide the ‘illusion of a global moral community’ (Fassin 2013, 37) even while they enact a division between the sufferers and their purported saviours. In an ironic illustration of staggering inequalities, superstar celebrities often stand in the limelight in these moral dramas; in this ‘postdemocratic’ political landscape, the power to judge who is worthy of help leaks into elite hands (Kapoor 2012). Speaking of the collusion of pop stars, entrepreneurs, politicians and civil society in contemporary humanitarianism, Mascarenhas argued that ‘the core of capitalism and the spirit of humanity are now one and the same … [T]he discourse of doing good has proved to be a powerful vehicle for reformulating the role not only of civil society groups but of governments and the private sector as well’ (Mascarenhas 2017, 139).

Imaginaries of the ‘Finnish welfare state’ and ‘Finnishness’ have transformed over time. For the second half of the 20th century, a key aspect of Finnish national identity was the belief that the state, rather than charities, should be the main provider of basic social services. Yet, in recent decades in Finland as in other Nordic contexts, tax-payers have increasingly begun seeing services that were earlier considered the responsibility of the state as morally acceptable recipients of charitable support (see, eg, Vamstad and von Essen 2013). The view of Finland as a ‘modern, functional welfare state’ (Valaskivi 2016, 142) has been consciously cultivated in country-branding campaigns, as has that of Finland’s exceptionally high-quality science. Yet the very idea of ‘Finnishness’ is also actively produced *through* science, in ways that sideline the notion’s racist and colonial foundations (Kuokkanen 2022). This is particularly so in the field of genetics (Oikkonen 2017; Tupasela 2016; Tupasela 2022), where imaginaries of Finnishness are constantly forged through the practices of biobanks (Tarkkala 2019)—a field of research and business in which Finnish hospitals play a vital part. Indeed, advertisements enrolling children to donate samples to Finnish biobanks (Snell and Tarkkala 2021) regularly feature on waiting room screens at the completed NCH. Yet, as we discuss below, the NCH’s entanglement in the global processes of biomedicalisation (Clarke *et al* 2010) were already evident in the NCH fundraiser.

One means for conceptualising these transformations is offered by the notion of assemblage. In thinking along the lines of ‘assemblage ethnography’ (Wahlberg 2022), we conceive of the NCH as emerging from a multiplicity of different lines: the ‘constantly changing components, (including) money, expert knowledge, time, specialist intervention’ (Youdell and McGimpsey 2015, 138) that converge in an assemblage. Assemblage thinking allows us to ‘connect localised, nontotalisable situations to broader political economies’ (Wahlberg 2022, 138), with a particular sensitivity to change over time. In a recent overview, Duff (2023) centres attention on the temporality of assemblages of health:

[T]he assemblage has a distinctive temporal dimension, a unique duration, insofar as the assemblage emerges in time in the accretion of constituent elements (human and nonhuman, intensive and extensive, affective and material), binding all such elements in relations of dependency as they act together as a condition of their mutual arrangement. (Duff 2023, 5)

Building on the call for more attention to be paid to how particular assemblages persist over time (Latour 2005), Duff highlighted the ‘forces of power and affect’ (Duff 2023, 5) that sustain assemblages of health.

Considering affects as ‘an effect of social processes that have worked to make them materialize’ (Martin 2013, 157), rather than as separated from cognition and subjectivity, we are interested in tracing how Finnish citizens’ moods (see Ssentongo and Alava 2023) were shaped and in turn shaped the assembling of a new hospital for sick Finnish children. The fact that the case in question is a hospital—a massive infrastructure project—is significant. Anthropologists have echoed Benjamin (1999) to posit that infrastructures ‘encode the dreams of individuals and societies and are the vehicles whereby those fantasies are transmitted and made emotionally real… [t]hey (are) embodiments of objective historical forces, but they simultaneously enter into our unconscious and hold sway over the imagination’ (Larkin 2013, 333). Hospitals are excellent examples of affective infrastructures, as they are, to use Sara Ahmed’s notion, often ‘sticky, or saturated with affect’ (Ahmed 2004, 11). Williams (2020), for instance, analyses the ‘Tommies’ hospital in London as ‘an unstable site for identity to attach its affects’, while Joanna Kehr discusses the ‘specific affective, economic and legal regimes’ that come together in hospital infrastructures (Kehr 2021). Describing how the construction of a pandemic hospital diverted attention from Spain’s austerity-induced healthcare crisis, Kehr uses Naomi Klein’s concept of ‘disaster capitalism’ (Klein 2007) to illustrate how the building of ‘spectacular’ hospitals can push the privatisation of public assets.

In the NCH case, the spectacle of the mould-infested and crumbling hospital (see Cityportal 2019) was accentuated by the campaign’s centring of children. As Ahmed (2004) argued, not all pain and suffering are rendered equal, nor do they create an equal emotive response in public or individuals. The figure of a child, as what Ahmed calls an ‘object of feeling’ (Ahmed 2004), holds a prominent position in the charitable imagination (Malkki 2015, 77). While the sentimental and economic value of children has changed enormously over time (Zelizer 1985), the recent century has seen children rendered, through charity campaigns, into suffering and vulnerable epitomes of humanity; children are routinely portrayed as ‘the exemplary human, and as politically harmless and neutral’ (Malkki 2015, 79), and, as such, particularly deserving of the empathy of potential donors (Bhati and Eikenberry 2016).

We propose thinking of both ‘Finnish children’ and the New Children’s Hospital built for them, as what Ahmed (2004) calls ‘objects of feeling’. As she argues, the ‘stickiness’ of emotions—their ascription to particular objects, be it sick children or a hospital—is produced by the erasure of the process that created them. For instance, the purported generosity of the donor towards the recipient diffracts attention from the original situation—the structures of inequality that necessitate it (Ahmed 2004, 28). In the NCH case, for instance, what is obscured by the charity campaign is the fact that the children’s hospital had been deprioritised until that date, not only by politicians, but ultimately by the population that had put those politicians in power and kept them there through multiple elections.

In this vein, we ask: what led Finnish people to become so invested in the figure of the suffering child and the charity-sponsored construction of a hospital to save her? Put otherwise, following assemblage theoreticians (Duff 2023; Latour 2005; Youdell and McGimpsey 2015): what brought the NCH assemblage together and made it stable? The key, we argue, is in what Ahmed describes as the emotional intensity that ‘sticks’ to some things more than it does to others. In the case of the NCH, this ‘stickiness’ was achieved through the way in which children and Finnish culture and society were portrayed in the NCH campaign and the debate that it triggered. Resonating with Ahmed’s analysis of a UK NGO’s letter to its donors, in the NCH campaign ‘generosity becomes a form of individual and possibly even national character; something “I“ or “we“ have, which is “shown” in how we are moved by others’ (Ahmed 2004, 22). Applying Ahmed’s terminology, children are exceptionally ‘sticky’—as this special issue contends.

## Methods and data

Our interest in the NCH was sparked by the call for papers to the symposium from which this special issue emerged. At the core of our analysis are three sets of data: first, and most importantly, 75 articles from five selected Finnish print media dating from the time of public debate around the NCH’s funding spree; second, a documentary published in 2019 about the NCH (Cityportal 2019); and third, the advertisement that launched the fundraising campaign, of which we conduct a close reading. Our interpretations of these data are enriched with interviews and informal discussions held by each of the authors in the contexts of our ethnographic research in Finland, which relate to paediatric pain care (Alava), physiotherapy (Lindroos) and organisations providing social help (Pihlman). These ethnographic studies inform our analysis of the NCH in terms of content, but they also shape our analytical approach, wherein we have considered different types of data as well as moments of ethnographic observation as equally relevant to an open-ended, explorative and improvisational process of data production and analysis (Cerwonka and Malkki 2007).

Driven by our basic interest in the relationship between children’s hospitals and charity, we focused on two major media: the privately owned daily newspaper *Helsingin Sanomat* (published by Sanoma News), and the publicly owned national broadcaster Yle. After performing an initial search in these media, we broadened the search to three media with more specifically focused readerships, whose views on the topic were of interest in the NCH case. These were *Lääkärilehti* (a newspaper published approximately twice a month by the Finnish Medical Association), *Kauppalehti* (a financial newspaper) and *Tekniikka & Talous* (a magazine focusing on technology and innovations, both published by Alma Talent Oy).

Informed by the timeframe of the NCH campaign, our search covered the period from January 2010 to December 2018, with keyword searches for ‘children’s hospital’ (*lastensairaala*), ‘new children’s hospital’ (*uusi lastensairaala*) and ‘children’s clinic’ (*lastenklinikka*). Using the newspapers’ online archives, available either directly on their website or through library repositories, we searched for all references to the children’s hospital in these media. Our dataset includes the 75 newspaper articles, opinion pieces and editorials we found published on the topic during this time, in which the questions of funding, decision-making or charity connected with the NCH are central concerns, rather than, for instance, progress in the technical process of its construction. The start of the public fundraising campaign in 2013 generated active discussions on the topic during that year, and out of the 75 pieces, as many as 50 documents were from 2013. The discussion significantly decreased in 2014 when the fundraising goal of 30 million euros was achieved.

Our analysis of the news articles and documentary followed the lines of content analysis (Tuomi and Sarajärvi 2018) in an abductive mode (Earl Rinehart 2021), that is, tacking between the data, our knowledge from other contexts and the evolving theoretical framework for analysis. Lindroos compiled the data and imported it into Atlas.ti. At this point, each author familiarised themselves with parts of the data, and we held a shared data analysis session, where a preliminary code list was decided on. The code list was shaped both by the symposium’s call and by our personal and ethnographic knowledge of Finnish society and of the NCH campaign, which we had all witnessed as citizens. From these starting points, our initial codes targeted our central aims: to understand who was seen as responsible for the condition of the hospital, and the ways in which the welfare state was defined, defended or critiqued. We then reconvened to discuss the data and coding and narrowed down the number of codes from the preliminary stage. The initial coding led us to realise that explicit references to emotions, and heavily affective rhetoric, were prominent in the data; consequently, following lengthy discussions on where we should focus our attention in the data, we created the final coding scheme with 15 codes. Lindroos then coded the entire data set, leading to altogether 364 individual uses of the codes.

After sifting through the data in this manner, we decided to tease out what we thought of as lines of argument. Rather than analysing them for frequency, we analysed them for their distinctness and for the degree they reflected some of the central lines that came together in the NCH assemblage. From among the many that we initially identified, we constructed three that stood out as analytically productive: in each of them, the relationships between the medical industrial complex, the state and charity are articulated in interestingly different ways.

## Lines of argument in public debate about the NCH

### Argument 1: hospitals must be publicly funded and under democratic control

As described above, the funding and management policy of the NCH represented a departure from what at the time was considered the norm and, unsurprisingly, it provoked a lot of critical debate. The heart of the argument for most critics writing in the media we have analysed was that caring for the most vulnerable citizens, such as sick children, is a core responsibility of the welfare state, which must be funded through tax revenues rather than charity. One of the key players in the NCH campaign, chief physician Jari Petäjä, himself acknowledged that ‘the children’s hospital should have been built with tax funds years ago [as it is] the duty of a civilized state’ (HS Sunday, 30 March 2013). Yet it is notable that the overwhelming majority of critical texts in our data are opinion pieces. In contrast, almost none of the editorials or journalistic reports on the campaign and, later, the hospital’s construction, contain this line of argument.

Numerous critics compared the NCH to other causes for which money had been found in state coffers, such as foreign aid or cultural institutions. Yet the crux of the critique was spelled out in the following: ‘I will not participate in the children’s hospital fundraising, but I accept that funds collected from my taxes are allocated to it’ (HS Opinion, 18 February 2013). As the campaign got underway, many commentators remarked on it as a precedent that must not be repeated, since ‘the Finnish welfare society cannot be built on collections’ (HS Opinion, 10 June 2013); ‘donations cannot be the way a welfare state cares for disadvantaged children’ (HS Opinion, 1 August 2013); and ‘access to help should not depend solely on the goodwill of donors’ (HS Column, 24 August 2013). These authors portrayed children as vulnerable and in need of protection, and insisted that it should be the state that protects, on account of children’s rights—not elites on account of their feelings of charity.

Over the course of the fundraising, and as the construction of the new hospital got underway, critiques that were central to public debate early on in the campaign virtually disappeared (see also Uoti 2016, 17). What came to dominate debate was the claim that the old model had failed, and a new model—the NCH model—was needed.

### Argument 2: children are suffering because politicians failed, we need a new model!

The second line of argument in media discussion of the NCH campaign identified the dire state of the old Children’s Clinic as proof of the failure of traditional political process. In a line of reasoning typical of much of the data, the chief physician of the Children’s Clinic described private donations as a last resort: ‘We have tried traditional methods [lobbying for funds from politicians] for years, and time is running out. These buildings simply no longer suffice for this purpose’ (Yle, 13 February 2013). Repeatedly, the disrepair of the children’s hospital was seen as more than an isolated case; rather, it was symptomatic of bigger problems. Indeed, both sick children and the welfare state itself were portrayed as requiring rescue from outdated and dysfunctional political structures. In one typical example, the editor of *Helsingin Sanomat*, the nation’s largest daily and a sponsor of the NCH, wrote:

When public funds are insufficient for a new children’s hospital, the project is accelerated by a foundation composed of economic and societal influencers – the well-off elite… The initiative is commendable, but at the same time, it demonstrates how poorly the welfare state has succeeded in its core mission. If we want to restore the welfare state to help those most in need, renovation is needed. (HS Editorial, 10 April 2013)

Resonating with debates lively in Finland at the time (see Saltman and Teperi 2016), the editor went on to call for simplification of the multichannel healthcare funding system, reconsideration of what taxes could cater to, and for ‘better competition and entrepreneurship in social and healthcare services’ (HS Editorial, 10 April 2013). Indeed, at the heart of the proposed system renovation was the opening of the public sector to both charity and business. Yet, in most instances in our data where a ‘new model’ is called for, there is an almost devout non-explication of any neoliberal political agenda. Rather, the turn to new, more efficient alternatives is offered as a commonsensical technical fix, one which the chairman of the Finnish Paediatric Society suggested might also ‘expedite’ other public hospital construction projects, and in which it was unnecessary to see any ‘political-ideological monsters’ (Lääkärilehti, 19 April 2013).

The proposed solution to the problem was presented in a straightforward manner by campaign figurehead Anne Berner at its launch: ‘If we suppose that everyone gives five euros, we will have already reached our goal’ (Yle, 13 February 2013). Such simplistic and supposedly non-politico-ideological ways of framing the charity campaign are perfect examples of depoliticisation: the presentation of a deeply political process or decision as merely technical (Ferguson 1994). This is an example of a broader trend in Finnish political debate, where calls for saving or ‘rescuing’ the welfare state can be employed to advance the opposite political ends (Kettunen 2022, 1), in this case, the claim that the welfare state is best saved through neoliberalisation. Since the 1990s, Finland’s care economy has been rapidly marketised and financialised: responsibility for care has increasingly shifted from the state to individuals and private businesses, which has eroded the previous normative expectation of state-provided, tax-funded care (Karsio *et al* 2023). The claims at the heart of the NCH campaign were thus reflective of and, we claim, also contributed to speeding up the neoliberalisation of Finland’s care economy. In the NCH campaign, children, as exceptionally ‘sticky’ objects of feeling, played a central role in nudging this process forwards.

### Argument 3: Finland’s outstanding paediatric care and medical technology can be used at and exported through the NCH

A distinct voice emerging in our data is that of experts lobbying for the hospital as a site for testing, using and championing Finland’s medical technologies and high-quality paediatric care. These arguments gained space in the media sources we analysed once the immediate debate over whether or not a charity-funding model is acceptable for the NCH receded. Drawing on interviews with multiple such experts, a journalist described:

The new children’s hospital … aims to be at the forefront of Nordic children’s clinics. Paediatric care in Finland is of a high standard by international comparison and the modern hospital is expected to improve care and research. The children’s hospital is reaching abroad to bring as large a population base as possible within its sphere of influence. International competition for patients is intensifying as the EU opens the borders for healthcare. (HS Domestic, 19 April 2014)

The hi-tech hospital was conceived as a win-win opportunity for Finnish sick children, hospital staff and the Finnish economy and start-ups alike. One commentator listed relevant areas of Finnish excellence—‘architecture, interior design, lighting, clothing for staff and patients, ventilation, information and equipment technology, as well as quality care’—and went on to state that while the treatment of sick children was a measure of a welfare state, the hospital provided ‘a nearly unique opportunity to showcase our expertise and significantly increase the export of high quality products and services’ (HS Opinion, 12 October 2013).

In an extensive article published in a magazine focused on technology and economy, the NCH was described as a ‘testing ground and reference institution for new Finnish health technologies’ that would attract international visitors, ultimately boosting Finland’s growing health industry and its already positive trade balance. In the piece, technoscientific imaginaries loom large, and the new hospital is envisioned as ‘filled with robots and virtual technology’ (*Tekniikka & Talous*, 5 December 2014). The article quotes the chairperson of the NCHF, Anne Berner: ‘The possibilities are absolutely amazing … In the hospitals of the future, machines will clean and disinfect, distribute linens, and dispense medications. They will support young rehabilitation patients learning to walk. They will even entertain and soothe’ (*Tekniikka & Talous*, 5 December 2014).

From the perspective of children and families entering the NCH, these imaginaries appear to have come true. In the hospital’s cafeteria, alongside donated children’s books and information leaflets from patient organisations, there is a table with crayons and pictures of fish, which children can colour in and enter into a scanner, from which they leap onto the two-floor high virtual ‘aquarium’ that lines the hospital’s foyer.

In the documentary published after the hospital’s opening (Cityportal 2019), the narrative of technical prowess is even more prominent than it was during the campaign. In the documentary, images of hi-tech hospital equipment go hand-in-hand with elated music and descriptions of the NCH as a pinnacle of scientific and technological excellence, and a demonstration of Finnish paediatric care as world class. A similar narrative of excellence is at the centre of other NCH-funded initiatives as well, such as the national Paediatric Pain Centre, which has been housed at the NCH since its launch with 3 years of donation funding in 2021. Through the evoking of such imaginaries of a techno-utopian healthcare future, the NCH has been woven into a story of Finland as a technologically advanced Nordic welfare society (see Valaskivi 2016), one where it is not just the state, but the people, the elite and savvy tech start-ups who care for the suffering child.

## Affecting a hospital into being: cultural repertoires of Finnishness in the NCH campaign

In the above, we have analysed three lines of argument that knotted together in the NCH assemblage. As demands for tax-funded basic services receded, the demand for a hospital built on the basis of a new and better funding model, with the aid and for the benefit of Finnish companies, took the upper hand. This was achieved not just through appeal to political or economic arguments, nor even to a purportedly pragmatic need to care for suffering children, but by evoking deep-seated cultural values and ‘Finnishness’. This was particularly clear in the fundraiser’s promotional material, which circulated through print, television and social media, and won numerous Finnish marketing and journalism awards (see Uoti 2016). To illustrate our claims, we turn our focus to the campaign’s launching advertisement, published pro bono on the front page of *Helsingin Sanomat* on 17 February 2013. In it, many of the elements that we see scattered across our data come into particularly sharp relief, and it thus serves as a perfect illustration of our argument. The advertisement begins with a story about buildings, buildings of all kinds, and of one building that is particularly needed but that has not yet been built—‘more precisely, its cornerstone hasn’t been laid’. The story then shifts tone, with its final lines thick with Finnish cultural references, which we underline in the following excerpt:

It’s a blatant sin, in the midst of all the abundance and well-being, that we haven’t yet managed to build a new children’s hospital. From this day forwards, we won’t accept any excuses. It’s time to straighten the curves. First, we’ll gather 30 million euros through public collection, then we’ll lay the foundation, hold a topping-out ceremony, and open the doors to those who need these walls and good care the most. It takes heart and *sisu* [roughly: ‘grit’]. Nothing more. The *talkoot* (collective work event) begin now and immediately. Welcome, join us. (HS front-page advertisement, 17 February 2013)

The advertisement shapes its pathos with a reference to ‘blatant sin’ (*suoranainen synti*), an expression that references the widely shared Lutheran heritage of Finns, and presumes that the audience understands and shares this reference. It then moves on to a well-worn phrase, ‘straightening the curves’ (*mutkat suoriksi*), a somewhat risky move, as the phrase connotes cutting through complexities or finding an efficient solution to a problem, but can also be used to criticise excessive simplification. The description of what will happen then ends in a word connoting a building’s topping-out ceremony (*harjakaiset*), which harkens back to pre-industrial and agricultural Finnish idylls.

The pinnacles of cultural cliché make their appearance in the concluding sentences. First, ‘It takes heart and *sisu*. Nothing more.’ The advertising company could not have used a more stereotypically Finnish word than *sisu* with which to appeal to its audience. *Sisu*, a concept recently ‘scientised’ and marketised for international export (Alava and Wolf-Meyer n.d), is commonly translated as ‘grit’, yet often described as ‘untranslatable’. It is used to refer to what is believed to be a quintessentially Finnish characteristic, a ‘can-do attitude’ that enabled the tiny country of Finland to beat Russia and maintain its independence in the Winter War of 1939–1940; to win incredible Olympic medals; and to develop the nation from rags to the riches of the welfare state (Helminen 2024). Finally, ‘The *talkoot* begin now and immediately.’ The notion of *talkoot* refers to shared collective work events that are often organised with happy socialising to follow (Eräsaari 2023, 100). It ‘echoes nostalgic ideas of a strong nation and sense of belonging to a local community’ (Uoti 2016, 75), and according to Uoti’s analysis, appealing to it was at the heart of the NCH campaign’s success. The campaign refurbished this nostalgic concept to refer to the national community, much as the concepts of *talkoot* and *sisu* have been used to mobilise Finns during the country’s wars, post-war reconstruction, and more recently, the downsizing of social welfare mechanisms.

Clarke and Parsell wrote, that ‘to understand the resurgence of charity, we must take account of the broader definition of the social that it is being mobilized to help produce’ (Clarke and Parsell 2022, 314). The social being produced through the NCH campaign, or to use Warner’s (2002) concept, the public that it evoked, was shaped by nationalism, elitism and racism. Elitism is apparent in statements like that made by Anne Berner at the campaign’s outset, when she speculated that ‘[i]f… everyone gives five euros, we will have already reached our goal’ (Yle, 13 February 2013). Not only did the statement strike at the notion of progressive taxation, a cornerstone of the former Finnish welfare state, it also occluded the fact that the key actor in the NCH drama was *not* ‘everyone’, but rather ‘the economic elite [which] took the reins when politicians failed to initiate the children’s hospital’ (HS Sunday, 31 March 2013).

The evocation of community, in which a presumed ‘everyone’ is brought to the same ‘*talkoot*’ to work for a good cause, was particularly striking in the music video made for the campaign song (Uoti 2016, 63), for in it, as in all of the campaign materials we have found, the entire cast was white. This is unsurprising for a country where commitment to Whiteness as a political project is commonplace (Hoegaerts *et al* 2022), and where the racist and nationalist undertones of biomedicine are particularly stark (Kuokkanen 2022; Tarkkala and Tupasela 2018). While appealing to the best interests of Finnish children, and by basking in what Dean (2020) calls the ‘good glow’ of charity, the NCH campaign reproduced the shared cultural hierarchies that render some apparently more worthy of help than others.

## The end of debate: the NCH and reassembled healthcare in post-welfare Finland

The completion of the NCH marked a significant break from the past half century’s history of welfare in Finland, a country where, as in other Nordic countries, charity had for decades been deliberately pushed to the margins (eg, Wijkström 2011). Due to the success of the NCH campaign, in resonance with charity runs for hospitals in the first decades of the 1900s, charity again gained prominence as a viable provider of new health infrastructure and care-provisioning arrangements. These changes, we hold, represent a neoliberal restructuring of what we interpret as Finland’s national assemblage of health (Duff 2023), one in which the specific assemblage of the NCH stands out as an important part. As Clarke and Parsell contended:

Although charity is mobilized to help reassemble the social according to neoliberal ideals, neither charity itself, the values it enacts, nor the relations it entails are themselves inherently neoliberal. They are rather complex and multivalent social processes that have diverse historical and cultural resonances. (Clarke and Parsell 2022, 322)

By considering the NCH as an assemblage, it has been possible for us to tease out some of the ‘complex and multivalent social processes’ and ‘diverse historical and cultural resonances’ through which the social is reassembled (Clarke and Parsell 2022, 322). Charity funding for a children’s hospital, in this analysis, is not simply ‘inherently neoliberal’. Rather, multiple lines came together, among them neoliberal ideological currents, to shape the NCH. Entangled in the NCH assemblage were a range of tangible human and non-human elements: the fungi growing in the too-moist walls of the old hospital; the bodies of sick children; companies working in health technology; the social networks of the individuals who started lobbying for a charity solution to the problem. But equally entangled were elements of a more ephemeral nature, such as affectively loaded cultural symbols and imaginaries of the nation, and the histories that gave particular ideas—of the child, of charity—their particular tenor in the Finnish context. The affective weight of these cultural symbols, and their systematic employment by the campaigners, was what turned both sick children and the NCH as a proposed solution to their predicament into ‘objects of emotion’ (Ahmed 2004). The notions of ‘*talkoot*’, of ‘*sisu*’, of ‘heart’ and the fairytale-like story of Finland’s path from rags to riches afforded the campaign just the right amount of ‘stickiness’ to make the NCH—as an assemblage (Duff 2023; Latour 2005; Youdell and McGimpsey 2015)—come together and stay together without disassembling.

What remains to be considered are the longer term consequences of the NCH being assembled as it was. As we have described above, once the funding appeal reached its target and the campaign was proved a ‘success’, voices critical of the replacement of tax-funded public service provision by charity were pushed out of the public domain, and the campaign’s core message—that funding basic services for children through elite-led charity campaigns is a good ‘Finnish’ way—took over, even offering a way to save the welfare state. Subsequently, the position of the NCH, as a turning point for Finnish healthcare policy and as a quasi-return to a pre-welfare state social contract, was obfuscated.

Since its opening, the NCH has served as a self-congratulatory symbol of purported equal opportunities for all Finnish children, and as a branding tool for Finnish clinical research on the global health science market. The hospital’s beautiful architecture, the nationally prized artwork beautifying its corridors, even the excellent level of care it provides to its patients, serve to mask the fact that behind the story of the NCH there lies a history of systematic political decision making that allowed paediatric care to fall off the priority list of politicians.

This deprioritisation is widely recognised by clinicians encountered by Alava in a study on the network for paediatric pain care in Finland, the launching of which was funded by the NCHF. At an event where the earlier lack of funding for specialised paediatric pain services in Finland was discussed, a representative of the NCH described why the foundation existed: ‘Children’s issues are easily forgotten and pushed aside by ‘more urgent’ matters in healthcare and other organisations. Children don’t vote. There are fewer children, and there are service needs throughout society’ (Alava, Fieldwork notes).

A similar idea was reflected in many clinicians’ responses to Alava’s question of why it had taken so long for the pain care network to be established, and why it had only been made possible through a significant donation of seed money from the NCHF. As one paediatrician described, ‘In elections … people choose someone who serves their interests, whoever can mobilise the most people. And it’s not the children.’ Indeed, children are exceptional not only as appealing objects of charity, but as citizens without voter rights. This is a point clinicians also acknowledge, among them the paediatrician mentioned above, who stated: ‘I think that I am the children’s advocate, I’m supposed to raise issues and talk about them.’ Paediatricians routinely advocate on behalf of their patients, and for sufficient resources with which to care for them, within the health bureaucracies in which they work. Yet in the case of the NCH, in part following years of unsuccessful lobbying for funds from politicians, the advocacy efforts of committed clinicians were swept up in the ‘good glow’ (Dean 2020) of charity.

Clarke and Parsell have argued that the resurgence of charity in welfare states serves to depoliticise poverty; charity responds to the immediate concerns of the poor, rather than addressing the structural factors that cause poverty in the first place (Clarke and Parsell 2022). A similar argument can be made with regard to children’s health. The NCH campaign gripped the Finnish public’s attention with a spectacle of fundraising for a massively expensive site for the provision of the most expensive kind of tertiary healthcare. The politics of inequality and its effects on children’s health is less likely to draw similar attention. As Liina Sointu *et al* have shown, there has been growing concern among Finnish parents about the quality of public healthcare services—concern to which many of those families who have sufficient funds and are healthy enough to be eligible have responded by taking out private insurance (Sointu *et al* 2021, 242). Both the turn to charity and the rise in private insurance can be seen as different facets of the changing assemblage of paediatric healthcare.

In turning to consider the long-term effects of the NCH campaign, it is worth citing at length Duff’s Deleuzo-Guattarian description of assemblages of health:

A given assemblage of health may combine health and social care infrastructures, such as hospitals and local clinics, which express desires for wellbeing and the efficient distribution of health care services and supports… These modes of organisation, and the desires expressed within them, are relatively autonomous insofar as they are made relatively durable over time, meaning that they are somewhat independent of the explicit desires, objectives and policy preferences of individuals and groups at any given time. Health care infrastructures endure beyond the cycles of politics and the preferences of political coalitions after all, intact if not unchanged. (Duff 2023, 6)

The particular way in which the NCH was assembled—the specific lines that convened in its inception and building—have had an effect on the Finnish healthcare and political landscape that extends far beyond the individual campaign, and that persists far after it ended: in the material form of the new hospital; in the legal, charitable and financial institutions that constitute it; and in the taken-for-granted mindset that these institutional forms are the direction in which the assemblage of Finnish healthcare will reassemble in the future.

## Data Availability

Data may be obtained from a third party and are not publicly available.
